# Mast Cell Mediators: Their Differential Release and the Secretory Pathways Involved

**DOI:** 10.3389/fimmu.2014.00569

**Published:** 2014-11-14

**Authors:** Tae Chul Moon, A. Dean Befus, Marianna Kulka

**Affiliations:** ^1^Pulmonary Research Group, Department of Medicine, University of Alberta, Edmonton, AB, Canada; ^2^National Institute for Nanotechnology, National Research Council, Edmonton, AB, Canada

**Keywords:** granule, lipid body, exosome, lysosome, exocytosis, secretion

## Abstract

Mast cells (MC) are widely distributed throughout the body and are common at mucosal surfaces, a major host–environment interface. MC are functionally and phenotypically heterogeneous depending on the microenvironment in which they mature. Although MC have been classically viewed as effector cells of IgE-mediated allergic diseases, they are also recognized as important in host defense, innate and acquired immunity, homeostatic responses, and immunoregulation. MC activation can induce release of pre-formed mediators such as histamine from their granules, as well as release of *de novo* synthesized lipid mediators, cytokines, and chemokines that play diverse roles, not only in allergic reactions but also in numerous physiological and pathophysiological responses. Indeed, MC release their mediators in a discriminating and chronological manner, depending upon the stimuli involved and their signaling cascades (e.g., IgE-mediated or Toll-like receptor-mediated). However, the precise mechanisms underlying differential mediator release in response to these stimuli are poorly known. This review summarizes our knowledge of MC mediators and will focus on what is known about the discriminatory release of these mediators dependent upon diverse stimuli, MC phenotypes, and species of origin, as well as on the intracellular synthesis, storage, and secretory processes involved.

## Introduction

Human mast cells (MC) are often characterized by their ability to release a variety of important mediators with a diversity of biological activities (1). The regulated release of peptides, amines, lipids, and even some gases depends on several molecular pathways: a prominent one of which releases large dense core vesicles (granules) through regulated exocytosis (degranulation); other pathways depend upon *de novo* production of mediators and complex vesicle trafficking and recycling, including constitutive secretion, exosomal and endosomal pathways; and other secretory pathways that are not dependent upon vesicles or membrane-bound moieties [e.g., gases such as nitric oxide by diffusion (2), lipid mediators from lipid bodies]. Although research is providing important new insights, we understand remarkably little about how the mediators are sorted into these secretory pathways and differentially released (Tables 1 and 2). Unanswered questions include: how are these pathways similar/dissimilar; how are mediators sorted into various compartments (e.g., progranules, granules, lysosomes, secretory vesicles, and exosomes); which stimuli activate these secretory pathways, and which proteins are involved; how do MC selectively release different cargo given different stimuli?

**Table 1:** **Mediators stored in human mast cell granules and their sorting mechanisms**.

Mediator	Sorting mechanism(s)	Reference
Amines	Vesicular monoamine transporter (VMAT)-2-dependent	(64, 65)
	Serglycin proteoglycan-dependent electrostatic interaction	(66)
Histamine		(20, 28)
Polyamines		(71)
Proteoglycans	Unknown	
Heparin		(36, 37)
Chondroitin sulfates		(36)
Serglycin		(60, 66)
Proteases	^a^Serglycin proteoglycan-dependent electrostatic interaction	
Tryptases		(21–31)
Tryptase-α		
Tryptase-βI		
Tryptase-βII		
Tryptase-βIII		
Tryptase-γ		(25, 27)
Tryptase-δ		(26)
Chymase-1		(30)
Cathepsin G		(30)
Granzyme B		(31)
Carboxypeptidase A3		(29)
Lysosomal enzymes	^b^Fusion with secretory lysosome	
β-Glucuronidase		(20)
β-Hexosaminidase		(20)
Arylsulfatase		(20)
Cytokines	Unknown	
TNF	^c^Endosomal pathway	(32)
bFGF		(33)
IL-4		(34)
SCF		(35)

*^a^Human mast cells proteases may be sorted into granules by serglycin proteoglycan-dependent electrostatic interaction based on the mouse study (52)*.

*^b^Lysosomal enzymes in human mast cell granules may be sorted by fusion of secretory lysosome and/or late endosome shown in RBL-2H3 cells (see Figure 2) (42)*.

*^c^Human mast cells sort TNF into granules *via* endosomal pathway, but rodent mast cells do it *via* mannose-6-phosphate receptor (M6PR)-dependent pathway (see Figure 2)*.

**Table 2:** **Stimuli-selective mediator release from mast cells (some representative examples)**.

Stimulus	Mechanism	Mediators	MC Types	References
**DEGRANULATION AND *DE NOVO* SYNTHESIZED MEDIATOR RELEASE**				
Antigen	FcεRI	Histamine	BMMC^a^, RBL-2H3	(155–161)
		cysLTs, PGD_2_, cytokines, chemokines, NO, ROS	hPBDMC, LAD2, HMC-1, rat PMC	
Neuropeptides (substance P, CGRP, capsaicin, etc.)	NKRs	β-Hexosaminidase, cytokines, chemokines	LAD2, hPBDMC	(162)
		cysLTs, PGD_2_	BMMC	(163, 164)
		5-HT	Rabbit MC	(165)
Compound 48/80	MrgprX2	β-Hexosaminidase, cytokines, chemokines, PGD_2_	BMMC	(164, 166)
Cathelicidin	GPCR	Histamine	Rat PMC	(167)
		Cytokines, chemokines, PGE_2_, LTC_4_	LAD2, hPBDMC	(168)
Defensins	GPCR	Histamine	Rat PMC	(167, 169, 170)
		Cytokines, chemokines, PGD_2_, PGE_2_, LTC_4_	LAD2, hPBDMC	(168)
Pleurocidin	FPRL1 (GPCR)	β-Hexosaminidase, PGD_2_, cysLTs, cytokines, chemokines	hPBDMC, LAD2	(171)
A23187	Ca^2+^ ionophore	Histamine	huMC, hPBDMC	(161, 172)
		β-Hexosaminidase	HMC-1	(173)
		Cytokines	FLMC	(174)
Morphine, codeine	Opioid receptors	β-Hexosaminidase, cytokines, chemokines	hPBDMC, LAD2	(175, 176)
Monomeric IgE	FcεRI	Cytokines,	BMMC	(177–179)
		β-Hexosaminidase	RBL-2H3	
			hCBDMC	
Nerve growth factor	Trk receptor	Histamine, PGD_2_, PGE_2_ cytokines	Rat PMC, BMMC	(180, 181)

***DE NOVO* SYNTHESIZED MEDIATOR RELEASE WITHOUT DEGRANULATION^b^**				
Zymosan, PGN, LTA	TLR2	GM-CSF, IL-1β, cysLTs	huMC	(88)
	Dectin-1 receptor	ROS	BMMC	(182)
PolyI:C, viral particles	TLRs	Cytokines	huMC progenitor	(183)
			LAD2, HMC-1, hPBDMC, BMMC	(184)
			KU-812	(185)
LPS	TLR4, CD14	Cytokines, chemokines	BMMC^c^	(186)
SCF	F-actin polymerization	Cytokines	hPBDMC	(187)
	MAP kinase kinase 3	Cytokines	BMMC	(188)
Lectins (ex: galectins)	TIM-3	Cytokines	HMC-1	(189)

**DEGRANULATION WITHOUT *DE NOVO* SYNTHESIZED MEDIATOR RELEASE EXCEPT ROS^d^**				
Complement peptides (C3a, C5a)	Complement receptors	Histamine	Human skin MC	(190–192)
Insect venoms	Guanylate cyclase	Histamine	Rat PMC	(193)
Pollutants (i.e. acrolein)		Histamine, ROS	RBL-2H3	(194)
Persulfate salts		Histamine, ROS	LAD2, KU-812	(195)
Advanced glycation endproducts (AGEs)		Histamine, ROS	Rat PMC	(196)
UV radiation		Tryptase	Human skin MC	(197)
Particulates (sodium sulfite, titanium dioxide nanoparticles, silver nanoparticles)	Non-FcεRI-mediated	Histamine, ROS	RBL-2H3 Rat MC	(198, 199)(200)

**NEITHER DEGRANULATION NOR *DE NOVO* SYNTHESIZED MEDIATOR RELEASE EXCEPT ROS**				
IgG	FcγRI, RIIA, RIII	ROS	BMMC, rat PMC, hPBDMC	(201)
Mercuric chloride (HgCl_2_)		ROS	Rat PMC	(202)
Gold compounds		ROS	Rat PMC	(202)
D-penicillamine		ROS	Rat PMC	(202)
Mechanical stretch		??	RBL-2H3	(203)
Gamma radiation		??	BMMC, hPBDMC	(204)

*^a^BMMC, mouse bone marrow-derived mast cell; CGRP, calcitonin gene related peptide; cysLTs, cysteinyl leukotrienes; FLMC, fetal liver-derived mast cell; FPRL1, N-formyl-peptide receptor 1; GPCR, G-protein coupled receptor; hCBDMC, human cord blood-derived mast cell; hPBDMC, human peripheral blood-derived mast cell; huMC, human mast cell; MC, mast cell; MrgprX2, Mas-related G-protein coupled receptor member X2; NO, nitric oxide; RBL-2H3, rat basophilic leukemia-2H3; ROS, reactive oxygen species; rat PMC, rat peritoneal mast cell; TIM-3, T cell immunoglobulin and mucin domain-containing protein 3; TLR, toll-like receptor; Trk receptor, neurotrophic tyrosine kinase receptor*.

*^b^No detectable degranulation or very minimal degranulation detected at the time points and doses tested thus far*.

*^c^Reported in murine MC but not in human MC*.

*^d^None or minimal secretion of *de novo* synthesized mediators except ROS that have been tested thus far*.

Constitutive exocytosis occurs in the absence of discernable stimuli for trafficking of secretory vesicles to the plasma membrane and can occur throughout the lifetime of a cell (3). Regulated exocytosis occurs after a clearly defined stimulus, either through changes in the extracellular environment [temperature (4, 5), pH (6), radiation (7), or osmolarity (8)] or ligation of a cell surface receptor (9). The pathways that control constitutive and regulated exocytosis have been extensively studied using powerful tools in high-resolution microscopy, molecular biology and animal model systems, and some of the molecules involved have been identified.

The terms degranulation, secretion, and exocytosis are often used interchangeably but have subtle variations in meaning. Degranulation refers to the loss of or release of granules and is most often associated with MC and basophils, both of which are characterized by their large intracellular granules. Secretion involves the release of a substance from one place of containment to another, i.e., from a cell to its extracellular environment or a gland to the skin’s surface. Excretion is the elimination of a waste material from a cell or organ. Exocytosis is a process of cellular secretion or excretion in which substances contained in vesicles are discharged from the cell by fusion of the vesicular membrane with the outer cell membrane (10–12). MC exhibit all forms of these release events but MC are perhaps best known for their rapid secretion of granules (degranulation) that contain large stores of pre-formed mediators (9).

**Figure 1:**
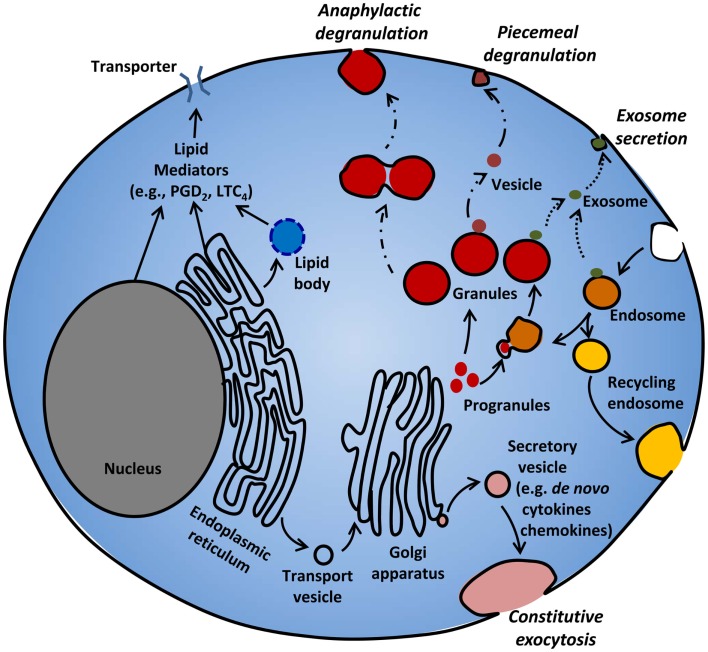
**Mediator release from MC**. MC release various mediators from different compartments following different stimuli. MC rapidly release pre-stored granule contents by piecemeal or anaphylactic degranulation. Immature progranules and mature granules can fuse with endosomes, and store lysosomal proteins. Some mediators can be released from granules and endosomes through exosomal secretion. Lipid mediators such as PGD_2_ and LTC_4_ are synthesized in lipid bodies, nuclear and ER membranes, and released through active transporters. *De novo* synthesized cytokines and chemokines packaged in secretory vesicles are released through constitutive exocytosis.

This review identifies our current understanding of the biogenesis of various mediator compartments, and the mechanisms of sorting and release of mediators from these compartments (Figure 1). We present some new postulates about exocytosis that may be particularly relevant to the MC, a highly specialized secretory cell (13). We also refer the readers some excellent recent articles for more details on various aspects of this subject (9, 14–19).

## Pre-Stored Mediator Release from MC Granules

### Mediators stored in MC granules

Mast cells are morphologically characterized by numerous, electron dense cytoplasmic granules which contain biogenic amines [histamine, serotonin] (20); several serine and other proteases {e.g., tryptase-α, -βI, -βII, -βIII, -γ [protease, serine S1 family member (PRSS) 31], -δ, chymase-1, cathepsin G, granzyme B, and carboxypeptidase A3} (21–31); lysosomal enzymes [β-glucuronidase (20), β-hexosaminidase (20), arylsulfatase (20)]; some cytokines [TNF (32), bFGF (33), IL-4 (34), and SCF (35)]; and proteoglycans [heparin (36, 37), chondroitin sulfates (36)] (Table 1). MC are able to overcome the large thermodynamic hurdle of storing high concentrations of these mediators in their granules by trapping them in an anionic gel matrix composed of chondroitin sulfates and heparin (38).

Subtypes of human MC are distinguished by the presence or absence of different serine proteases in their granules (i.e., tryptase^+^/chymase^−^: MC_T_, tryptase^+^/chymase^+^: MC_TC_, and tryptase^−^/chymase^+^: MC_C_). MC activation has typically been measured by monitoring the release of granule mediators (degranulation), with a particular focus on histamine, β-hexosaminidase, or tryptase (39, 40). Pre-stored mediator release through MC degranulation can be an early and rapid event following stimulation, resulting in the release of large portions of stored histamine within 15–90 s. This release of pre-formed mediators enables not only rapid anaphylactic reactions and allergic responses but also initiates recruitment of leukocytes to sites of pathogen invasion, activation of innate immune processes, and inflammatory responses (1). Other longer term responses associated with granule-derived mediators include wound healing and tissue remodeling processes through multiple communications with other cells (e.g., fibroblast proliferation and extracellular matrix production by histamine and MC proteases) (41).

### MC granule heterogeneity and biogenesis

#### Heterogeneity

**Figure 2:**
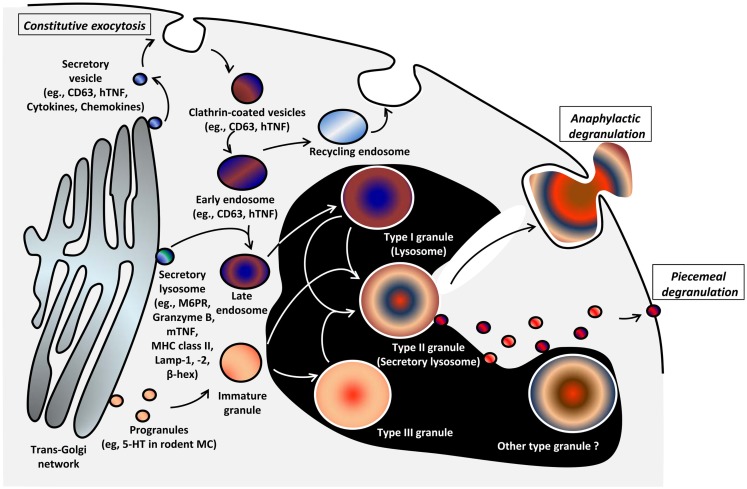
**Model of genesis of MC secretory lysosomes (granules) and their heterogeneity/plasticity [adapted from Raposo et al. (43)]**. Type I granules and type III granules are formed from lysosomal/endosomal pathway and by unit granule fusion from the trans-Golgi region, respectively. Secretory lysosomes that bud from trans-Golgi network contain MHC class II molecules, mannose-6-phosphate receptor (M6PR), and the lysosomal markers LAMP-1, -2, and β-hexosaminidase. It is postulated that post-endosomal, type II secretory lysosomes arise through the fusion of Type I and III granules. The relationship of this model to observations of heterogeneity of secretory lysosomes with regard to histamine or 5-HT content and VAMP-8 expression is unclear and there likely exists more granule heterogeneity/plasticity than three types (44). The mechanism of genesis of granule types is poorly understood (black area).

Mast cells granules, also called secretory lysosomes, contain both lysosomal proteins such as acid hydrolases, e.g., β-hexosaminidase, as well as mediators such as histamine, and can secrete both together. MC also contain traditional lysosomes that can release enzymes such as β-hexosaminidase independently of histamine (42). Raposo et al. (43) distinguished three types of granules in mouse bone marrow-derived MC (BMMC) based on their contents of MHC class II, the lysosomal marker β-hexosaminidase, lysosomal membrane protein (LAMP)-1, LAMP-2 and mannose-6-phosphate receptors (M6PR), and the biogenic amine, serotonin: type I granules contain MHC class II, β-hexosaminidase, LAMP-1, LAMP-2, and M6PR but not serotonin (perhaps a classical lysosome); type II granules contain MHC class II, β-hexosaminidase, LAMP-1, LAMP-2, M6PR, and serotonin (perhaps a late secretory lysosome); type III granules contain β-hexosaminidase and serotonin but not MHC class II (Table 3) (42, 43). Baram et al. proposed a model wherein type II granules are generated by fusion of type I and type III granules, which contain lysosomal proteins and secretory amines, respectively (42). However, there has been little experimental follow-up of this postulate and there is evidence that MC have more diverse types of granules than depicted by this model (Figure 2). Indeed, the relationship between this classification of granules and observations that serotonin and cathepsin D vs. histamine and TNF exist in distinct granule populations (see below) in mouse MC is unclear (44). It is likely that MC granules are more heterogeneous than the three types shown above (Figure 2; Table 3) and that this heterogeneity may depend on the tissue of residence and the species, health status, and even age of the individual (1, 45).

**Table 3:** **Mast cell secretory granule subsets**.

	Contents	Associated proteins	Reference
Type I	Cathepsin D	LAMP-2	(44)
	β-Hexosaminidase		(42)
		MHC-II	(43)
		M6PR	(72)
		LAMP-1 and 2	(43)
Type II	Histamine		(44)
	Serotonin	VAMP-8	(42, 44)
	β-Hexosaminidase		(42, 44)
		MHC-II	(43)
		M6PR	(72)
		LAMP-1 and 2	(43)
Type III	TNF (may be in type II as well)		(44)
	Serotonin	VAMP-8	(42, 44)
	β-Hexosaminidase		(42, 44)
		M6PR	(72)

#### Biogenesis

The biogenesis of MC granules involves regulated fusion of what are called unit granules (small fusogenic granules) (46). These early unit granules buds from the trans-Golgi region and fuse to generate progranules in a region delimited by the outermost Golgi cisternae, rough endoplasmic reticulum (ER), and mature granules in the cytoplasm. The volumes of progranules are multiples of unit granules (i.e., volume of progranule created by three unit granules is three times unit granule volume). Progranules leave this zone as immature granules and become mature through a fusion process with other immature or mature granules. A process called “condensation” reduces the granule volume and organizes the contents, generating various sizes of mature granules (15). In addition to the homotypic fusion, which is postulated to form type III granules, immature granules or type III granules are also able to fuse to endosomes or lysosomes (Type I granules) in what might be the pathway that forms type II granules (secretory lysosomes) as Baram et al. proposed (see above and Figure 2) (42, 43). However, MC granules are likely more heterogeneous than the three types postulated, and our understanding of the later phase of granule biogenesis is thus depicted inside a black area in Figure 2.

#### Molecules involved in MC granule biogenesis

Several proteins involved in MC granule biogenesis and maturation have been identified (Table 4).

**Table 4:** **Molecules that are (or may be) involved in mast cell granule biogenesis and homeostasis**.

Protein	Function	Cell type	Reference
**DEMONSTRATED IN MAST CELLS**			
Histidine decarboxylase	Promotes granule maturation	BMMC	(54)
Synaptotagmins	Membrane-trafficking	RBL-2H3	(42)
Secretogranin III	Regulates membrane dynamics of secretory vesicles via interaction with chromogranin A	RBL-2H3	(51)
Chromogranin A	Binds secretogranin and promotes granule biogenesis	RBL-2H3	(51)
Clathrin	May be involved in compensatory endocytosis following exocytosis and granule recycling	Mouse peritoneal MC	(205)
Polyamines	Regulate granule cargo storage and granule morphology	BMMC	(71)
Vesicular monoamine transporter 2 (VMAT2)	Transport of monoamines into secretory granules	Mast cells, megakaryocytes, thrombocytes, basophils, and cutaneous Langerhans cells from patients with mastocytosis	(206)
Serglycin	Retention of proteases in granules	BMMC	(52)
Nuclear receptor 4a3	Modifies granule contents	BMMC	(207)

**DEMONSTRATED IN OTHER CELL TYPES BUT MORE WORK NEEDED IN MAST CELLS (POSSIBLE NEW PATHWAYS)**			
V-ATPase	Hyperacidification of lysosomes	Many cell types	(208)
AP-1A	Transports cargo between the trans-Golgi network and endosomes	Corticotrope tumor cells	(209)
Rabs (32 and 38)	Trafficking enzymes into vesicles	Melanocytes	(210)

##### Rab GTPases

Rab3d and Rab5 play roles in the fusion of immature granules. MC from Rab3d knockout mice have granules that are larger than in MC from wild-type mice (47), while knockdown of endogenous Rab5 or expression of constitutively negative mutants of Rab5 significantly reduces the size of granules and increases their number (48). Moreover, Rab5 plays a role not only in homotypic granule fusion (type III granule biogenesis) but also in granule/endosome heterotypic fusion (type II granule biogenesis), and vesicle associated membrane protein (VAMP)-8 is involved in Rab5-mediated fusion of granules.

##### Lysosomal trafficking regulator

Chediak–Higashi syndrome, a mutation of the lysosomal trafficking regulator (*LYST*) causes the formation of giant granules in many cells including MC, and can be studied using the orthologous lyst-deficient beige (*Lyst^bg^/Lyst^bg^*) mouse. In MC and pancreatic acinar cells of beige mice, there is giant granule formation, presumably the result of disordered fusion of granules, suggesting Chediak–Higashi syndrome (CHS)/*Lyst* plays a role in controlling granule fusion (15, 49).

##### Synaptotagmins

The synaptotagmins (Syts) are membrane-trafficking proteins with at least 15 members in mammals. The RBL-2H3 MC line expresses Syt II, III, V, and IX (42) and RBL-2H3 treated with antisense to Syt III showed enlarged granule size, impairment in granule maturation, and formation and delivery of internalized transferrin to the perinuclear endocytic recycling compartment involved in a slow recycling pathway (50).

##### Granin family

The granin family of proteins, first described in neuroendocrine cells, are also important in MC granule biogenesis and maturation. Secretogranin III, for example, is present in MC granules, depleted during MC degranulation and over-expression of this protein causes an expansion of the secretory vesicle compartment (51). Although the biologic role of this family of proteins is not well understood, they are involved in cholesterol sequestration, interact with chaperones of granule proteins, serve as precursors of granule cargo and function as calcium buffering proteins, making them potentially intriguing players in the life history of MC granules. Is it possible that some of their many functions are necessary to control progranule fusion and thereby aid their maturation of secretory lysosomes/granules? Could these proteins somehow regulate the core components of the fusion machinery and thereby determine which progranules fuse together? Further experimentation is required in this area.

##### Histamine and proteoglycans

Some granule mediators themselves such as histamine and the core proteoglycans such as serglycin are also important components of the granule maturation process. For example, BMMC from serglycin knockout mice have functional secretory granules but they are defective in dense core formation (52). Furthermore, these serglycin^−/−^ BMMC are resistant to apoptosis associated with reduced release of proteases and defective caspase-3 activation (53). In addition, the lack of histamine or the enzymes that control its synthesis significantly alters the morphology and contents of granules. Peritoneal MC from histidine carboxylase knockout mice show abnormal granule morphology and contain fewer proteases and heparin (54). This is mainly due to the down-regulation of genes encoding granule proteases and enzymes involved in heparin biosynthesis. Interestingly, agonists of the H4 histamine receptor and exogenous application of histamine restored granule maturation. Therefore, histamine likely influences early steps in granule maturation but has little role in maintaining the integrity of fully formed mature granules since depletion of histamine from mature MC had no obvious effect on granule structure. It would be interesting to examine the effect of histamine depletion on immature MC (i.e., from CD34^+^ progenitors) as they progress through the MC differentiation process.

##### Adaptor-protein family

Components of our models of MC granule biogenesis can be extrapolated from other cells with complex secretory granule processes, such as pituitary lactotropes, pancreatic β-cells, and transfected tumor cells. In these cells, progranules in the trans cisterna of the Golgi are covered with clathrin coats, which contain the adaptor-protein (AP) family of proteins that can bind cytosolic tails of transmembrane protein cargo, facilitating their entry into budding vesicles (55). Immature secretory granules are not responsive to secretagogues and it appears that one of the essential roles of the AP proteins is to facilitate their maturation (56). Could APs perform a similar function in MC? Clearly, additional studies are needed to further understand the genesis, heterogeneity, and plasticity of MC granules and uncover potential therapeutic opportunities within such knowledge; a frontier.

### Mechanisms of sorting and storage of pre-formed mediators in MC granules

The most unique and igneous feature of MC granules is their ability to store large concentrations of mediators in a small space for long periods. In theory, collecting a high concentration of such highly charged mediators in a membrane-enclosed space would require a large amount of osmotic work and create a thermodynamic disadvantage. However, MC trap the mediators in an anionic gel matrix composed mainly of heparin and chondroitin sulfate, which confers a huge thermodynamic advantage (38, 57). Upon cell activation, the polymer gel phase undergoes a transition and swells to release the mediators (58). This efficient solution is not-surprisingly conserved among living organisms and even phytoplankton use a similar packaging and degranulation process (59). Current understanding of sorting and storage mechanisms of pre-formed mediators are reviewed below and in Table 1 but it is still poorly understood.

#### Proteoglycans

Proteoglycans, a core component of MC granules are heavily glycosylated proteins, consisting of a core protein and glycosaminoglycan side chains that are covalently attached to the core through glycosidic bonds. In MC, serglycin is a dominant core protein, and heparin and chondroitin sulfate are dominant glycosaminoglycans that can be used to distinguish some MC subpopulations (1). Sulfation of glycosaminoglycans imparts a negative charge on the proteoglycan, which is an important mechanism that helps to retain proteases and biogenic amines in MC granules (60). An ion exchange mechanism with the charged glycosaminoglycans is thought to be significant in mediator release from granules (61, 62). Moreover, as outlined above, proteoglycans are important in granule composition and maturation. However, details of the sorting mechanism of proteoglycans and other mediators into MC granules are poorly understood. Studies with rat pancreatic acinar cells provide clues and suggest that glycosaminoglycan side chains are necessary for proteoglycan sorting into granules, as deletion of serine–glycine repeat region of the serglycin core protein and treatment with p-nitrophenyl-β-D-xylopyranoside, an alternate substrate for glycosaminoglycan side chain attachment, prevented sorting into granules and lead to accumulation of proteoglycans in Golgi (63).

#### MC proteases

Mast cell proteases are synthesized in the ER, modified in the Golgi complex, and sorted into progranules that bud from the trans-Golgi. Retention of MC proteases in granules depends on the serglycin proteoglycan in murine MC, as absence of mouse MC protease (mMCP)-5 (chymase) and carboxypeptidase, and reduction of mMCP-6 (tryptase) occurs in the granules of MC from serglycin deficient mice despite normal expression of protease mRNA and granule formation (52). Although limited information is available with other kinds of proteases, serglycin proteoglycan is involved in certain protease retention in MC granules. However, the mechanisms underlying the trafficking of MC proteases into the granule need to be elucidated and confirmed in human MC, although the storage of heparin and chondroitin sulfate proteoglycan in human MC granules has been shown (36).

#### Biogenic amines

Histamine and serotonin are biogenic amines stored in MC granules. There is evidence that transport of biogenic amines from cytosol into the MC granules occurs in a vesicular monoamine transporter 2 (VMAT2)-dependent manner (64, 65). Moreover, retention of biogenic amines and release from the granule is serglycin proteoglycan dependent (66). Whether retention of both MC proteases and biogenic amines are directly dependent on serglycin proteoglycan or involve only the glycosaminoglycan side chains, e.g., heparin, with their electrostatic charges (67–70), needs to be elucidated and extended to studies with human MC. Some polyamines (such as putrescine, spermidine, and spermine) are required for granule homeostasis and possibly aid in the native conformation and packaging of other granule molecules such as histamines and proteases (71).

#### Lysosomal enzymes

Many lysosomal enzymes (Table 1) are found in MC granules but the detail mechanisms of their sorting, trafficking, storage, and secretion are poorly understood. It is postulated that they are transported into type II MC granules when granules and endosomes fuse (Figure 2). Clearly, lysosomal enzymes can be found in both type II granules, as well as classical lysosomes (type I granules), and secreted from both compartments. Using MC from serglycin knockout mice, it was shown that storage and release of β-hexosaminidase is independent of serglycin (52).

#### Cytokines

Among the large number of cytokines and chemokines released after MC activation, TNF (32, 72), bFGF (33), IL-4 (34), and SCF (35) are known to be pre-stored in MC granules, and can be released by regulated exocytosis, as well as synthesized following MC activation and released through constitutive exocytosis (Figure 1) (73, 74). Many other cytokines and chemokines appear not to be stored [e.g., GM-CSF (75)], but are newly synthesized following MC activation and are secreted by constitutive exocytosis over the course of several hours/days (discussed below) (9, 76). For storage in the granule, there appears to be a different trafficking mechanism for TNF in rodent and human MC. In rodent MC, sorting of TNF from ER to granules occurs *via* a brefeldin A- and monensin-sensitive route, utilizing a M6PR-dependent pathway and N-linked glycosylation of asparagine at N86 (Figure 2) (72). By contrast in human MC, TNF does not utilize this pathway as the N-linked glycosylation motif NSS of rodent TNF is replaced by the RTP motif (32). By transfecting and chasing fluorescence-tagged TNF into human MC lines (HMC-1 and LAD2), Olszewski et al. showed that in human MC, TNF traffics to the plasma membrane transiently, but then is stored in MC granules by endocytosis (Figure 2) (32). These observations emphasize that evidence acquired from studies of rodent MC must be validated for human MC, as has been shown for several other examples of species differences among MC (1). Apart from TNF, the mechanisms of trafficking of bFGF, IL-4, and SCF, another cytokines stored in human MC granules have not been studied, and we still do not fully understand how these cytokines are sorted in granules and which secretion pathway(s) initiates their release. Based on the binding affinity of bFGF for heparin (77), the retention mechanism of bFGF in the granule is likely to be heparin dependent, although this needs to be confirmed. Immunohistochemistry has shown that IL-4 but not IL-5 are stored in MC secretory granules in the lung parenchyma and nasal mucosa of patients with active allergic rhinitis (34). Although the amount of IL-4 in the granules increases after FcεRI-mediated activation, it is unclear whether the majority of IL-4 released extracellularly is due to degranulation or constitutive exocytosis.

### Mechanisms of secretion of pre-formed mediators from MC granules

Two types of degranulation have been described for MC: piecemeal degranulation (PMD) and anaphylactic degranulation (AND) (Figures 1 and 2). Both PMD and AND occur *in vivo*, *ex vivo*, and *in vitro* in MC in human (78–82), mouse (83), and rat (84). PMD is selective release of portions of the granule contents, without granule-to-granule and/or granule-to-plasma membrane fusions. PMD in MC has been identified in numerous settings, ranging from chronic psychosocial stress (85) to estradiol (86), CCL2 (87) and TLR stimulation (88), and interactions with CD4^+^/CD25^+^ regulatory T cells (89). The granule morphology is relatively well retained following PMD, although ultrastructural changes are evident (79). It has been proposed that the mechanism of PMD involves the budding of vesicles containing selected mediators from granules and their transport to the plasma membrane, fusion, and mediator release (Figures 1 and 2) (90). Little is known about the molecular machinery involved in these processes.

In contrast to PMD, AND is the explosive release of granule contents or entire granules to the outside of cells after granule-to-granule and/or granule-to-plasma membrane fusions (Figures 1 and 2). Ultrastructural studies show that AND starts with granule swelling and matrix alteration after appropriate stimulation (e.g., FcεRI-crosslinking). Granule-to-granule membrane fusions, degranulation channel formation, and pore formation occur, followed by granule matrix extrusion (81). Granule-to-granule and/or granule-to-plasma membrane fusions in AND are mediated by soluble *N*-ethylmaleimide-sensitive factor attachment protein receptors (SNAREs) (91). In human intestinal MC, protein expression of soluble *N*-ethylmaleimide-sensitive factor attachment protein (SNAP)-23, syntaxin (STX)-1B, STX-2, STX-3, STX-4, VAMP-2, VAMP-3, VAMP-7, VAMP-8, and STX-6 have been reported (92). However, only VAMP-7 and VAMP-8 were found to translocate to the plasma membrane and interact with SNAP-23 or STX-4 upon activation. Moreover, inhibition of SNAP-23, STX-4, VAMP-7, or VAMP-8, but not VAMP-2 or VAMP-3, reduced histamine release mediated by FcεRI-crosslinking (92). Therefore, VAMP-7, VAMP-8, SNAP-23, and STX-4 are important SNARE molecules in human intestinal MC granule fusion and exocytosis.

#### Molecules involved in MC degranulation

Several proteins involved in MC degranulation were listed in Table 5.

**Table 5:** **Molecules involved in mast cell degranulation**.

Protein	Function	Cell type	Reference
Munc 13-4	Positively regulates degranulation	RBL-2H3	(93, 104)
Munc-18-2	Controversial in degranulation, interacts with syntaxin-3	RBL-2H3	(94, 95)
Complexin II	Enhances Ca^2+^ mobilization and degranulation	RBL-2H3	(96)
VAMP-8	Controversial in degranulation	BMMC, RBL-2H3	(44, 48)
Synaptotagmin II	Controversial in degranulation	BMMC, RBL-2H3	(42, 62, 99)
Rab3a	Controversial in degranulation	RBL-2H3	(101, 102)
Rab3d	Negatively regulates degranulation	RBL-2H3	(101)
Rab27a	Negatively regulates degranulation, regulates cortical F-actin integrity	BMMC, RBL-2H3	(93, 105)
Rab27b	Positively regulates degranulation	BMMC	(105, 106)
Rac1	Positively regulates degranulation	RBL-2H3	(107, 108)
Rac2	Positively regulates degranulation, regulates Ca^2+^ mobilization	BMMC	(109)
Cdc42	Positively regulates degranulation, interacts with PLCγ1, increases IP_3_ production	RBL-2H3	(107, 108)
DOCK5	Positively regulates degranulation, regulates microtubule dynamics, phosphorylation and inactivation of GSK3β	BMMC	(110)
MARCKS	Negatively regulates degranulation, delay of degranulation	BMMC, eHMC^a^	(111)

*^a^eHMC, embryonic hepatic-derived mast cells*.

##### Mammalian uncoordinated-18 proteins

The functions of SNARE proteins are regulated by several accessory proteins, but our knowledge is incomplete and at least in part, the information is controversial (Table 5). Munc 13-4 was shown to be a target of Rab27a and Munc 13-4-transduced RBL-2H3 release more histamine compare to the mock-transduced cells after IgE/Ag stimulation (93). In rodent MC, mammalian uncoordinated-18 (Munc-18)-2, located in the granule membrane, interacts with STX-3 and plays a role in granule-to-granule as well as granule-to-plasma membrane fusion (94, 95) whereas, Munc-18-3, located in the plasma membrane, also interacts with STX-4 (94). Following activation of RBL-2H3, another protein, complexin II translocates from the cytosol to the plasma membrane and interacts with a SNARE complex. Although translocation of complexin II to the plasma membrane did not induce membrane fusion, the reduction of degranulation after knockdown of this protein suggests that complexin II is a positive regulator of MC degranulation (96). The fusion of the SNAREs with the plasma membrane has been examined using transmission and freeze-fracture electron microscopy and biophysical modeling. About 30–60 s after activation, unetchable circular impressions about 80–100 nm in diameter were found on the E face (intracellular face) of the plasma membrane (97). These impressions are not permanent but are postulated to form the fusion sites for the granules directly preceding degranulation. Under certain conditions, these fusion sites can form rosettes and the coupling of this structure with the plasma membrane may then form a cup-shaped structure called a porosome (98). Due to the nanometer size of these SNARE docking sites, the MC degranulation complex has been called a nano-machine (97).

##### Vesicle associated membrane proteins

A study using BMMC from VAMP-8 deficient mice showed reduced serotonin, cathepsin D, and β-hexosaminidase release, but normal histamine and TNF release following IgE-mediated or PMA/ionomycin stimulation (44). By contrast, transfection of VAMP-8 in RBL-2H-3 did not affect the calcium ionophore/12-*O*-tetradecanoyl-13-acetate- or IgE-mediated release of fluorescent-labeled neuropeptide Y, which is stored in the same granules as serotonin and β-hexosaminidase (48). These conflicting data may be the result of different experimental systems (former using deficient mouse of VAMP-8 and latter using over-expression), or the fact that the latter study examined mediator release indirectly with fluorescent-labeled neuropeptide Y. Although further study is required, VAMP-8 is likely involved in granule biogenesis and degranulation of subsets of MC granules, which contain serotonin, cathepsin D, and β-hexosaminidase. Moreover, this suggests that there is heterogeneity of MC granules (Figure 2), and that distinct mechanisms are involved in mediator release between subsets of granules.

##### Synaptotagmins

Synaptotagmin (Syt) II depresses Ca^2+^-triggered secretion of β-hexosaminidase and MHC class II release (42), but increases cathepsin D release in RBL-2H3 and mouse BMMC (99). Moreover, in Syt II knockout mice there is a marked deficiency in degranulation and an impaired passive cutaneous anaphylaxis response (62). In RBL-2H3, Syt IX can regulate protein export from the endocytic recycling compartment to the plasma membrane and play a role in sorting proteins of secretory granules (100). Much remains to be learned about these proteins and MC function.

##### Rab GTPases

In addition to their role in granulogenesis mentioned above, the Rab family of GTPases is also involved in MC degranulation. Over-expression of Rab3a in RBL-2H3 showed no (101), or inhibitory effects (102) on FcεRI-mediated β-hexosaminidase release, while over-expression of Rab3d demonstrated that it translocates from the granule to the plasma membrane (103), and inhibits degranulation (101). Recently, it was established that Rab27a is located in histamine-containing granules in RBL-2H3 and that over-expression of constitutively active Rab27a reduced FcεRI-mediated histamine secretion (93). Munc 13-4 was found to be a target of Rab27a, and the Rab27a-Munc 13-4 complex was required for docking of granules to the plasma membrane and release of granule contents in RBL-2H3 cells (104). Moreover, Rab27a regulates cortical actin stability with its effectors melanophilin (Mlph) and myosin, as well as Rab27a/b/Munc13-4-dependent granule exocytosis (105). Rab27b knockout mice exhibited reduced passive cutaneous anaphylaxis and defects in FcεRI-mediated β-hexosaminidase release from BMMC (106). Although Rab5 is involved in MC granule biogenesis (see above), a transfection study showed that Rab5 is not involved in granule mediator release (48).

##### Rho GTPases

Among Rho GTPases, Rac and Cdc42 play a positive role in RBL-2H3 degranulation by regulating IP_3_ production, upstream of Ca^2+^ influx and interacting with PLCγ1 (107, 108). More recently, the roles of Rac1 and Rac2, which have a 92% sequence identity, in MC degranulation were dissected using knockout mice (109). In BMMC from Rac2 knockout mice, FcεRI-, but not Ca^2+^ ionophore-mediated β-hexosaminidase release was defective because of a decrease in Ca^2+^ flux without changing F-actin remodeling and membrane ruffling, which are regulated by Rac1 (109).

##### Others

The cytoskeleton and the microtubule network is an essential component of the degranulation process in MC. Proteins such as DOCK5 (110), MARCKS (111), and myosin VI (112) regulate the progress of secretory granules through the cytoskeletal network and allow them to dock with the plasma membrane. When these proteins are disrupted, the degranulation process does not occur normally. Some of these pathways remain unexplored in MC, yet we know that some microtubule events facilitate granule fusion and are essential to degranulation. For example, myosin Va forms a complex with Rab27a and Mlph thereby regulating cortical F-actin stability upstream of Rab27a/b/Munc13-4-dependent granule exocytosis (105).

Although calcium flux is unequivocally an essential feature of the degranulation process [recently reviewed by Fahrner et al. (113) and Ashmole and Bradding (114)], other ion exchange complexes have also been implicated, such as potassium and chloride channels that facilitate and regulate Ca^2+^ signaling. Certainly, disruption of membrane potentials using cationic liposomes can impair Ca^2+^ flux and suppress the function of SNAP-23 and STX-4 (115). It has been suggested that inhibitors of these ion exchange pathways may be useful in the treatment of inflammatory diseases that are mediated by MC activation and degranulation (116).

Calcium flux is essential for degranulation but its role may be more extensive than first postulated and some theories have questioned the long-standing belief that granule membranes must fuse with the plasma membrane to facilitate exocytosis. There are new theories of exocytosis that have not yet been examined in MC, including the theory of porocytosis, or secretion without membrane fusion, in which Ca^2+^ ions form salt bridges among adjacent lipid molecules through which mediators would move according to mass action (13). This quantal secretion theory has been postulated to be important in neuromuscular junctions and the central nervous system and it offers an intriguing process for mechanisms underlying constitutive exocytosis. However, this mathematical model has not been validated experimentally in neurons or other secretory cells. Autophagy, an evolutionarily conserved bulk degradation system that facilitates the clearance of intracellular molecules, has also been shown to be an important regulator of MC exocytosis. A recent study by Ushio et al. has shown that proteins that normally control autophagy may also facilitate the fusion of small secretory vesicles and facilitate their fusion with the plasma membrane (117). In fact, many new regulatory pathways have been connected to degranulation and exocytosis. However, we need more sophisticated model system to validate these new theories. Given that most molecules known to be involved in MC degranulation have been studied in rodent models, their roles in human MC must be examined and potential distinctions between MC phenotypes in different species should be investigated.

## Secretion of Exosomes

Exosomes are membrane-bound vesicles of ~30–100 nm that appear to bud from the internal surface of multivesicular bodies in the endosomal compartment of many cell types including MC (Figure 1) (118). They are important in cell–cell communications and a breadth of physiological and pathophysiological responses, notably antigen presentation and host defenses. The contents of exosomes include a richness of lipids such as ceramide, cholesterol, phosphatidylserine, and sphingomyelin; a great diversity of proteins (200–400) including MHC class II, phospholipases, heat shock proteins, co-stimulatory molecules (CD40, CD40L, and CD86), adhesion molecules, kinases, tetraspanins, cytoskeletal proteins, chaperones, aldolase A, TNF, FcεRI chains, processed peptides from antigens; and a plethora of mRNA (>1800) and microRNA (>100) species (119–122). Exosomes transfer diverse cargo and functional capacity among cells; for example, mRNAs and microRNAs in MC exosomes can be transferred between human and mouse MC or other cells and control gene expression in recipient cells (119, 120). Carroll-Portillo et al. emphasized the potential functional significance of IgE-antigen and antigenic peptide–MHC class II MC–T cell–Dendritic cell interactions, as well as MC uptake of antigen-crosslinked receptor (16).

Mast cell exosomes can be secreted by both constitutive or regulated exocytosis (Figure 1) (16, 43) and the composition of protein constituents differs depending upon which pathway is employed (16). Knowledge is advancing rapidly about the molecular bases of exosome biogenesis, regulation of exosome loading, and the secretory pathways involved. The reader is referred to recent reviews of this intriguing and rapidly evolving subject (118, 123).

## Cytokine/Chemokine Secretion from Compartments Other than Secretory Lysosomes

In addition to regulated secretion of a limited repertoire of cytokines from stores in granules (above) through pathways of PMD or AND, MC secrete a diversity of cytokines and chemokines by other pathways [diagrammed by Lorentz et al. (14) in their Figure 3], including: constitutive exocytosis, better known in macrophages [e.g., Ref. (3, 9), etc.], and exosomal secretion (Figure 1). Frank et al. showed that involvement of SNAREs in chemokine release of human intestinal MC using their specific neutralizing antibodies. They showed that CXCL8, CCL2, CCL3, and CCL4 release after IgE/α-IgE stimulation were abrogated by inhibition of STX-3 or SNAP-23, but not by inhibition of STX-2 or VAMP-3. Moreover, inhibition of different SNARE subsets selectively reduce chemokine release (i.e., STX-4 or VAMP-8 inhibition selectively reduce CXCL8, and STX-6 inhibition reduces CXCL8 and CCL2) (124). However, other study using VAMP-8^−/−^ BMMC showed that VAMP-8 does not affect cytokine/chemokine secretion (125). Therefore, there has been insufficient exploration of the molecular elements involved in these pathways in MC; careful analyses are needed in comparison to other cells such as macrophages, as well as among various MC subsets and between human and other MC.

## Lipid Mediator Release

Activated MC release an abundance of arachidonic acid metabolites, notably leukotriene (LT) C_4_, prostaglandin (PG) D_2_, and platelet activating factor (PAF) (126–128). These lipid mediators have bronchoconstricting and vasoactive properties, but also participate in host defense, inflammation, and allergic diseases through diverse activities such as effector cell trafficking, antigen presentation, immune cell activation, and fibrosis (129–131). Eicosanoids, lipid mediators derived from arachidonic acid, are *de novo* synthesized and released immediately from activated cells rather than stored, and exert autocrine and paracrine functions. However, recent observations of intracellular localization of the PGD_2_ receptor, DP2/CRTh2, suggest intracrine functions of this mediator (132, 133). Eicosanoid synthesis can occur at several sites in the cell, including the ER (134), nuclear membrane (134, 135), phagosomes (136), and cytoplasmic lipid bodies (Figure 1) (81, 82, 137, 138), and the site of synthesis likely depends on the cell type, as well as the nature of the stimulation [see review in Ref. (139)]. Whether the enzymes and other proteins needed for eicosanoid synthesis are *de novo* synthesized following cell activation, or are dispersed in the cell and then translocated to a site of synthesis following activation, are under debate. In either case, how these proteins are targeted to a specific intracellular compartment of eicosanoid synthesis is unknown.

### Lipid bodies

**Figure 3:**
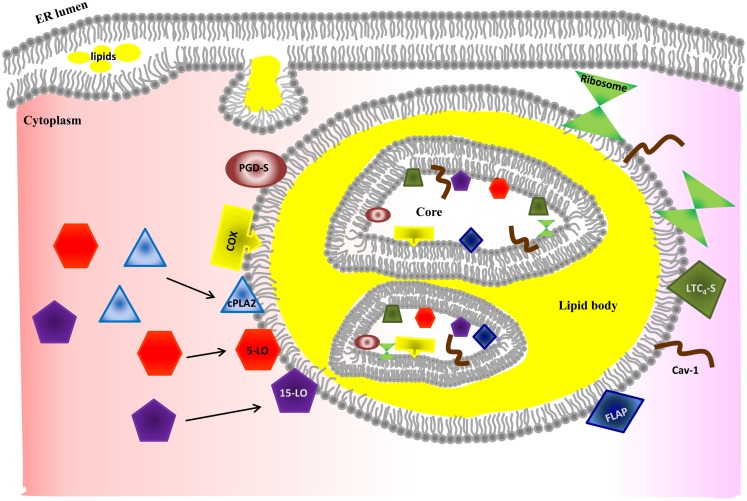
**Model of lipid body biogenesis and structure**. Neutral lipids synthesized in the ER accumulate between bilayer of ER membrane and bud off as a lipid body. Lipid bodies have phospholipid monolayer on their outside. However, lipid bodies contain a bilayer core structure inside, which provides a hydrophilic area. The bilayer core can be created by incorporation of multiple loops of ER membrane and explains how ER membrane proteins (e.g., caveolin-1 and ribosome) are incorporated into lipid bodies. However, the exact mechanism of formation of the bilayer structure is poorly understood. Enzymes required for eicosanoid production have been found in both outer membrane and core of lipid bodies. Increased intracellular Ca^2+^ after MC stimulation induces activation and translocation of cPLA_2_, 5-LO, and 15-LO to the lipid body membrane for eicosanoid synthesis. Further studies are required to unveil how MC control synthesis and secretion of arachidonic acid metabolites in a stimulus-specific fashion.

Lipid bodies also referred to as lipid droplets are osmiophilic organelles surrounded by a monolayer of phospholipids and containing lipids with a unique fatty acid composition. Lipid bodies are composed of a core rich in neutral lipids and a variety of proteins depending upon the cell type and the conditions of stimulation (Figure 3) (137, 138, 140). These organelles are one of the major sites of eicosanoid generation. They are inducible during inflammatory processes and increase in size and number in several types of leukocytes, including MC (141–144). The numbers of lipid bodies in the cytoplasm of MC differ among MC phenotypes. For example, lipid bodies are rare in human skin MC in comparison to numbers in lung and gut MC (81, 82). In addition to eicosanoid synthesis, a few cytokines and chemokines are also found in lipid bodies of eosinophils (TNF), neutrophils (TNF), macrophages (TNF), and MC (TNF and bFGF) (33, 142). Much remains to be elucidated about the kinds of cytokines stored in lipid bodies, functions of these cytokines, and the mechanisms of their release.

### Lipid body biogenesis

Although the details of the biogenesis of lipid bodies in MC are poorly understood, they bud from the ER membrane where neutral lipids are synthesized (Figure 3) (145). Lipid bodies can also increase in size by fusion and this process occurs in a rapid regulated way (146).

### Mechanisms of synthesis and secretion of lipid mediators

Eicosanoids are made by oxidation of 20-carbon fatty acids. After activation, MC synthesize PGD_2_ and LTC_4_ from sequential enzymatic reactions of arachidonic acid metabolism of cyclooxygenase and lipoxygenase pathways, respectively. PAF also can be synthesized from lysophosphatidylcholine by PAF-acetyltransferase. Both arachidonic acid and lysophosphatidylcholine can be generated from membrane phospholipid (1-O-alkyl-2-arachidonoyl-*sn*-glycero-3-phosphocholine) by phospholipase A_2_. Recently, studies on pro-resolving lipid mediators, such as resolvins, protectins, and maresins from eicosapentaenoic acid (EPA) and docosahexaenoic acid (DHA) have been advanced in macrophages and our understanding of their physiological, pathophysiological functions in resolution of inflammation are improved, but little is known their synthesis in MC (147).

Because eicosanoids synthesized intracellularly are negatively charged at physiological pH (148), they diffuse poorly across membranes. Thus, their secretion involves active transport through either organic anion transporters of the ATP-binding cassette type C family, also known as multidrug resistance-related proteins (149–152), or organic anion transporters of the solute carrier superfamily (153, 154). However, little is known of these mechanisms in MC and further study is needed to identify the mechanisms and their regulation.

## Conclusion

Over several decades, there have been significant advances in our knowledge of MC biology that have transformed our understanding of this multifaceted immune cell from an effector cell in allergic inflammation to a major player in innate and acquired immunity. Because of the pivotal role of MC in allergic and other inflammatory reactions, therapeutic strategies to disrupt the action of MC mediators have been developed and used widely [e.g., anti-histamines, anti-IgE (omalizumab), and Cys-LT1 receptor antagonist (montelukast)]. However, there remain large gaps in our knowledge about intracellular trafficking of MC mediators, particularly in the selective mechanisms of storage and secretion that are often dependent on the specific stimuli involved. Our knowledge of the biogenesis of MC cargos, the heterogeneity of granules, and the molecules involved in trafficking pathways of mediator secretion are rapidly evolving and likely to be a productive in helping to unravel the complexities of MC biology. A greater understanding of mediator specific trafficking pathways will provide opportunities to develop novel therapeutic targets for the treatment of increasingly wide-spread allergic and other inflammatory diseases.

## Author Contributions

Tae Chul Moon, A. Dean Befus, and Marianna Kulka equally contributed to writing this paper.

## Conflict of Interest Statement

The Guest Associate Editor Paige Lacy declares that, despite having collaborated with authors Tae Chul Moon and A. Dean Befus, the review process was handled objectively and no conflict of interest exists. The authors declare that the research was conducted in the absence of any commercial or financial relationships that could be construed as a potential conflict of interest.

## Abbreviations

AND, anaphylactic degranulation; BMMC, bone marrow-derived mast cell; ER, endoplasmic reticulum; LAMP, lysosomal membrane protein; MC, mast cell(s); M6PR, mannose-6-phosphate receptor; PMD, piecemeal degranulation; SNAP, soluble *N*-ethylmaleimide-sensitive factor attachment protein; SNARE, soluble *N*-ethylmaleimide-sensitive factor attachment protein receptor; STX, syntaxin; Syt, synaptotagmin; VAMP, vesicle associated membrane protein.
